# Predictive factors for the surgical treatment of necrotizing enterocolitis in preterm infants: a single-center retrospective study

**DOI:** 10.1186/s12887-021-02973-w

**Published:** 2022-01-03

**Authors:** Yusheng Liu, Lingyan Qiao, Xiongwei Wu, Zhong Jiang, Xiwei Hao

**Affiliations:** 1 Department of Pediatric Surgery, The Affiliated Hospital of Qingdao University, Qingdao, 266000 Shandong Province China; 2 Department of Pediatric Endocrinology and Genetic Metabolic Diseases, Qingdao Women and Children’s Hospital, Qingdao, 266000 Shandong Province China

**Keywords:** Necrotizing enterocolitis, Premature birth, Risk factors, Surgery

## Abstract

**Background:**

Necrotizing enterocolitis (NEC) is a gastrointestinal disease that tends to occur in premature infants. Some features may be associated with an increased probability that preterm infants with NEC will require surgical treatment. This study aimed to identify the factors that increased the probability of surgical treatment in infants with NEC.

**Methods:**

We retrospectively analyzed the data of premature infants with NEC who were hospitalized at The Affiliated Hospital of Qingdao University from April 2011 to April 2021. According to the treatments received, these patients were divided into medical NEC group and surgical NEC group. The perinatal characteristics, clinical manifestations, and laboratory values before the onset of NEC were subjected to univariate and multivariate analyses.

**Results:**

A total of 623 preterm infants with NEC (> Bell’s stage I) were included in this study, including 350 (56%) who received surgical treatment and 273 (44%) who received conservative medical treatment. Multivariate analysis showed that lower gestational age (*P* = 0.001, odds ratio (OR) (95% CI) = 0.91[0.86–0.96]), early occurrence of NEC (*P* = 0.003, OR (95% CI) = 0.86 [0.77–0.95]), hemodynamically significant patent ductus arteriosus (*P* = 0.003, OR (95% CI) = 7.50 [2.03–28.47]), and low serum bicarbonate (*P* = 0.043, OR (95% CI) = 0.863 [0.749–0.995]) were associated with an increased probability of surgical treatment in preterm infants with NEC.

**Conclusions:**

Our findings were applied to identify potential predictors for surgical treatment in preterm infants with NEC, which may facilitate early decisive management.

## Background

Necrotizing enterocolitis (NEC) is a serious gastrointestinal disease of the neonatal period that is especially common in premature infants [1]. With the increasing availability of neonatal intensive care units and technical improvements, the survival rate of preterm infants is increasing yearly, and the incidence of NEC is also gradually increasing [2]. The reported incidence rate of NEC in the United States is 0.07–0.1% in general and reaches 5–10% in very low birth weight infants, and approximately 30–50% of NEC patients require surgical treatment [3].

The slow and insidious onset and the nonspecific clinical manifestations of NEC make an accurate diagnosis difficult. For clinicians, the diagnosis of NEC is mostly based on the observation of nonspecific systemic inflammatory signs and abdominal signs combined with radiography. Even the widely accepted pathognomonic features of pneumatosis and portal venous gas occur late in disease and can be subject to interobserver variability [4]. To date, there is still controversy regarding which clinical manifestations and laboratory tests can be used as potential predictors of the clinical deterioration of NEC. Therefore, our goal is to determine which perinatal characteristics, clinical manifestations, and laboratory test results are associated with an increased probability of receiving surgical treatment in preterm infants with NEC.

## Methods

### Subjects

We retrospectively analyzed the medical records of preterm infants (gestational age < 37 weeks) treated in the Affiliated Hospital of Qingdao University who were diagnosed with NEC during the period from April 2011 to April 2021. NEC (> Bell’s stage I) was diagnosed based on clinical manifestations and imaging findings [5]. To determine which factors are related to the severity of NEC and the increased probability of receiving surgical treatment in preterm infants, we collected perinatal data, clinical manifestations, and laboratory test results before the onset of NEC and the relevant results during medical or surgical treatment.

All medical records were collected independently by two experts, and consensus was reached through consultation in case of disagreement. Based on treatment, the patients were divided into a medical necrotizing enterocolitis (mNEC) group and a surgical necrotizing enterocolitis (sNEC) group. The indications for surgery were obvious intestinal perforation (pneumoperitoneum) on radiographs or suspected intestinal necrosis or intestinal perforation that was not confirmed by radiographs when the clinical symptoms were not improved after long-term conservative treatment. The presence of pneumoperitoneum was confirmed by preoperative radiography or intraoperative exploration and pathological examination results. Patients who had surgical indications but could not undergo surgical treatment due to the severity of their condition were assigned to the sNEC group. Patients in the sNEC group were excluded if they were diagnosed with solitary perforation during exploratory laparotomy and pathological examination. Surgical methods included abdominal drainage, exploratory laparotomy, intestinal resection and anastomosis, and enterostomy.

The study was conducted in accordance with the Declaration of Helsinki and this study was approved by the Ethics Committee of The Affiliated Hospital of Qingdao University (Project identification code: QDFY-KY-2021-012). Given the retrospective nature of this study, the ethics committee approved the waiver of parents’ written consent.

### Statistical analysis

SAS v 9.4 (SAS Institute, Carly, NC) was used for the statistical analysis. A 2-tailed *P* < 0.05 was considered statistically significant. First, frequency distributions and descriptive statistics were retrieved, and the distribution of the data was assessed for normality by applying a Shapiro-Wilk test. Continuous variables are expressed as median (interquartile range) and were analyzed with the Mann-Whitney test. Categorical variables are expressed as numbers (%) and were analyzed with the chi-squared test, Cochran-Armitage trend test, or 2-tailed Fisher’s exact test. Univariate and multivariate logistic regression analyses were performed to identify the surgical risk factors for necrotizing enterocolitis. The results are presented as two-tailed *p* values, odds ratios (OR), and corresponding 95% confidence intervals (95% CI).

## Results

### Pre- and perinatal variables

A total of 623 preterm infants with NEC were included in this study, including 340 males and 283 females with an average gestational age of 29 + 2 weeks (interquartile range = 24 + 6-35 + 5) and an average birth weight of 1105.2 ± 272.8 g. A total of 162 patients were diagnosed as stage IIA, 60 were diagnosed as stage IIB, 222 were diagnosed as stage IIIA, and 179 were diagnosed as stage IIIB. The corrected gestational age at the onset of NEC was 31 + 1 weeks in the mNEC group and 29 + 5 weeks in the sNEC group. A total of 350 (56%) patients received surgical treatment, and 273 (44%) patients received conservative medical treatment. There were 42 patients who had surgical indications but were determined to be unsuitable for surgical treatment due to the presence of surgical contraindications. These 42 patients eventually died of NEC. The mortality rate was 10% (17 out of 162) for stage IIA NEC, 0% (0 out of 60) for stage IIB, 65% (145 out of 222) for stage IIIA, and 47% (85 out of 179) for stage IIIB. The mortality rate was 8% (23 out of 273) in the mNEC group and 64% (224 out of 350) in the sNEC group. Compared with NEC patients who received only conservative medical treatment, lower gestational age (*P* = 0.001) and lower birth weight (*P* = 0.015) were positively correlated with an increased probability of receiving surgical treatment. Multivariate analysis showed that for every increase of 1 day in gestational age at birth, the risk of receiving surgical treatment decreased by approximately 9% (Table 1).

**Table 1:** Pre- and perinatal characteristics of surgical and medical NEC patients

	Surgical NEC (*n* = 350)	Medical NEC (*n* = 273)	*P*	Unadjusted OR [95%CI]	*P*	Adjusted OR [95%CI]
Gestational age (median [IQR] weeks+days)	28 + 1 [24 + 6-33 + 3]	30 + 4 [27 + 1-35 + 5]	0.001	0.92 [0.87–0.96]	0.001	0.91 [0.86–0.96]
Birth weight (mean [SD], g)	1014.9 [207.2]	1264.9 [256.8]	0.015	0.997 [0.995–0.999]		
Gender male (n [%])	187 [53.4]	153 [56.0]	0.825	0.90 [0.36–2.28]		
Delivery mode vaginal delivery (n [%])	247 [70.6]	136 [49.8]	0.073	0.41 [0.16–1.09]		
Multiple birth (n [%])	153 [43.7]	102 [37.4]	0.581	1.30 [0.51–3.36]		
PPROM (n [%])	68 [19.4]	25 [9.2]	0.239	2.34 [0.57–9.67]		
Meconium amniotic fluid (n [%])	0 [0]	17 [6.2]	0.999	NA		
1-min Apgar (median [IQR])	5 [3–8]	5 [2–6]	0.360	1.10 [0.90–1.342]		
5-min Apgar (median [IQR])	8 [7–9]	7 [6–8]	0.834	1.03 [0.80–1.31]		
Mother’s age (mean [SD])	29 [4]	28 [6]	0.717	1.02 [0.92–1.12]		
Antibiotics during pregnancy (n [%])	68 [19.4]	76 [27.8]	0.390	0.62 [0.21–1.85]		
Antihypertensive medication (n [%])	25 [7.1]	57 [20.9]	0.160	0.34 [0.08–1.52]		
Corticosteroids (n [%])	256 [73.1]	238 [87.2]	0.141	0.39 [0.11–1.37]		
Magnesium sulfate (n [%])	85 [24.3]	119 [43.6]	0.084	0.42 [0.15–1.13]		
Oxytocin antagonist (n [%])	68 [19.4]	76 [27.8]	0.390	0.62 [0.21–1.85]		

*IQR* Interquartile range, *SD* Standard deviation, *n* Number, *PPROM* Prelabor rupture of the membrane

### Clinical variables

Age at the onset of NEC (*P* = 0.007), hemodynamically significant patent ductus arteriosus (hsPDA) (*P* = 0.007), the occurrence of late-onset sepsis within 72 h before the onset of NEC (*P* = 0.022), and failure to achieve complete enteral nutrition before the onset of NEC (*P* = 0.003) were associated with an increased probability of receiving surgical treatment. Multivariate analysis of the above factors showed that the diagnosis of NEC 1 day earlier increased the probability of receiving surgical treatment by approximately 14% and that hsPDA increased the pediatric patients’ probability of receiving surgical treatment by 650% (Table 2).

**Table 2:** Clinical features for surgical and medical NEC patients

	Surgical NEC (*n* = 350)	Medical NEC (*n* = 273)	*P*	Unadjusted OR [95%CI]	*P*	Adjusted OR [95%CI]
NEC onset days (median [IQR])	10 [7–15]	15 [9–21]	0.007	0.90 [0.83–0.97]	0.003	0.85 [0.77–0.95]
Time until first defecation days (median [IQR])	2 [1–3]	2 [1–4]	0.716	0.95 [0.72–1.26]		
Late onset sepsis < 72 h prior to clinical onset (n [%])	204 [58.3]	85 [31.1]	0.022	3.11 [1.18–8.21]		
Hemodynamically significant PDA (n [%])	187 [53.4]	59 [21.6]	0.007	4.14 [1.46–11.69]	0.003	7.60 [2.03–28.47]
RBC transfusion days (median [IQR])	1 [1–2]	1 [0–3]	0.619	0.92 [0.67–1.27]		
Mechanic ventilation exposure (n [%])	256 [73.1]	153 [56.0]	0.134	2.12 [0.79–5.67]		
Mechanic ventilation days (median [IQR])	4 [1–6]	3 [0–9]	0.767	1.01 [0.92–1.11]		
Enteral feeding type (n [%])						
Breast milk	139 [39.7]	112 [41.0]	0.346	Reference		
Formula milk	42 [12.0]	8 [2.9]	0.211	4.29 [0.44–41.95]		
Combination	169 [48.3]	153 [56.1]	0.682	0.80 [0.28–2.28]		
Achieved full enteral feeding (n [%])	128 [36.5]	170 [62.3]	0.030	0.35 [0.13–0.90]		
Total days parenteral feeding (median [IQR])	9 [5–13]	9 [5–12]	0.894	1.01 [0.90–1.13]		
Postpartum antibiotics (n [%])						
Not administered	25 [7.1]	76 [27.8]	0.081	Reference		
1–3 days administered	187 [53.4]	119 [43.6]	0.038	4.71 [1.09–20.47]		
> 3 days administered	138 [39.5]	78 [28.6]	0.033	5.33 [1.14–24.90]		
Antibiotics days (median [IQR])	5 [3–9]	6 [3–8]	0.844	0.99 [0.89–1.11]		
Medication (n [%])						
Antimycotics	76 [21.7]	51 [18.7]	0.737	1.22 [0.38–3.87]		
Corticosteroids	42 [12.0]	25 [9.2]	0.703	1.34 [0.30–6.09]		
Inotropes	128 [36.6]	51 [18.7]	0.100	2.50 [0.84–7.45]		

*IQR* Interquartile range, *n* Number, *RBC* Red blood cell, *PDA* Potent ducts arteriosus

### Laboratory variables

The results of laboratory examinations performed within 72 h before the onset of NEC showed that low platelet count (*P* = 0.019), low serum bicarbonate (*P* = 0.003), and high base excess (*P* = 0.004) were associated with an increased probability of receiving surgical treatment in NEC patients. Multivariate analysis of the above results showed that for every 1% decrease in the serum bicarbonate level of NEC patients, the probability receiving surgical treatment increased by 14% (Table 3).

**Table 3:** Laboratory values of NEC patients in the study

	Surgical NEC (*n* = 350)	Medical NEC (*n* = 273)	*P*	Unadjusted OR [95%CI]	*P*	Adjusted OR [95%CI]
Leucocytes× 10^9^/L (median [IQR])	20.4 [10.8–29.2]	17.2 [10.3–32.9]	0.945	0.998 [0.952–1.047]		
Hemoglobin mmol/L (mean [SD])	7.9 [1.06]	8.1 [1.7]	0.656	0.92 [0.62–1.35]		
Platelet counts×10^9^/L (median [IQR])	146 [91–267]	272 [177–389]	0.019	0.995 [0.990–0.999]		
Arterial blood gas (mean [SD])						
PH	7.18 [0.10]	7.23 [0.12]	0.058	0.00 [0.00–1.23]		
PCO_2_ KPa	7.63 [1.83]	8.05 [3.07]	0.562	0.93 [0.73–1.18]		
PO_2_ KPa	4.91 [1.95]	4.23 [3.62]	0.734	0.96 [0.76–1.22]		
HCO_3_^−^ mmol/L	20.35 [4.92]	26.71 [6.84]	0.003	0.83 [0.74–0.94]	0.043	0.863 [0.749–0.995]
Base excess mmol/L	−8.0 [5.02]	−1.6 [7.2]	0.004	0.85 [0.76–0.95]		
Lactate mmol/L	1.91 [0.73]	2.74 [1.39]	0.145	0.42 [0.13–1.35]		

*SD* Standard deviation, *CRP* C-reactive protein, *NEC* Necrotizing enterocolitis, *OR* Odds ratio, *IQR* Interquartile range, *HCO*_*3*_^*−*^ Bicarbonate

## Discussion

NEC is an idiopathic inflammatory disease characterized by extensive hemorrhage and necrosis of the small intestine and colon. It is the leading cause of emergency gastrointestinal surgery during the neonatal period and is a leading cause of death in preterm infants between 2 weeks and 2 months of age [6, 7]. In clinical practice, NEC can be controlled in approximately half of infants after fasting, antibiotic treatment, and supportive treatment. However, some children require surgical intervention due to the progressive aggravation of the disease. Therefore, early recognition of the clinical manifestations of NEC deterioration and timely diagnosis and treatment are extremely important to reduce the mortality of NEC.

Similar to the results of some previous studies, our study found that children with a lower gestational age and birth weight were more likely to receive surgical treatment. Many scholars have also reached similar conclusions. Bazacliu et al. [8] found that the morbidity and mortality of NEC were inversely proportional to the gestational age. In a 2-year national study in the United Kingdom, the incidence of NEC was 11% in infants born at 24 weeks of gestation but decreased to 0.5% in infants born at 31 weeks of gestation [9]. Hull et al. [10] found that the probability of receiving surgery in children with NEC decreased with increasing birth weight, namely, for every 100 g increase of birth weight, the probability of undergoing surgery was reduced by approximately 5%. In a study on the incidence rates of NEC in infants of different races, the authors found that the proportion of infants with preterm birth and low birth weight was higher in Hispanic and non-Hispanic black infants; thus, they speculated that premature birth and low birth weight, rather than ethnic factors, led to the higher incidence of NEC [11]. These findings can be explained by the immature intestinal development of preterm infants. The gastrointestinal tract supports the immune system and nervous system, which may be a determinant of intestinal injury and inflammation in infants with NEC. Pathogenic microorganisms invade the immature intestinal tract, resulting in increased morbidity and mortality. In addition, many infants with NEC achieve a high volume of enteral feeding at the time of onset, but the increase in intestinal volume may provide more substrate for the growth of pathogenic microorganisms and place greater pressure on the intestine. In contrast, the development of gut microbiota in preterm infants differs from that in full-term infants [12]. Studies have shown that preterm infants have lower gut microbial diversity, different colonized microbes in the intestine, and more potential pathogenic bacteria [13, 14]. A multivariate analysis of the above factors showed that only lower gestational age was an independent predictor of the incidence of sNEC.

In our study, we found that NEC occurred earlier in children who required surgical treatment than in those who required only medical treatment. This result was confirmed by Duci et al. [15]. The intestinal tract matures gradually over time after birth, which may reduce the probability of undergoing surgery in infants with severe NEC. There is controversy regarding whether there is a correlation between patent ductus arteriosus (PDA) and the occurrence of NEC. It has been reported that compared with NEC patients without PDA, NEC patients with PDA have a better prognosis [16]. However, another study found that the presence of PDA increased the mortality of NEC patients [17]. In addition, extremely low birth weight infants have a significantly higher incidence of PDA, which is commonly treated with nonsteroidal anti-inflammatory drugs (NSAIDs). Another study has shown that the incidence and severity of NEC are related to the use of NSAIDs [18]. In our study, we found that many children with NEC had received NSAIDs to treat hsPDA, which was found to be related to an increased probability of receiving surgical treatment. We speculated that the deterioration of the condition of NEC patients with hsPDA might be due to the decreased intestinal perfusion and decreased oxygenation caused by hsPDA. Multivariate analysis showed that clinical early-onset NEC and hsPDA that required treatment were associated with an increased probability of surgical treatment in NEC patients and were independent predictors for sNEC.

In some studies, sepsis has been identified as a risk factor for NEC [19, 20]. The onset of NEC can be likened to that of early onset sepsis, which is rapid and fatal and has serious consequences for patients. Studies have shown that the incidence of NEC in children with sepsis is almost 3 times that in children without sepsis [21]. Antibiotics are the most commonly used drugs in neonatal intensive care units, especially for extremely premature infants. The use of antibiotics reduces the biological diversity of gut microbiota, delays the colonization of beneficial microbes, and promotes the excessive growth of pathogenic bacteria. At least 3 cohort studies have found that the empirical use of antibiotics for more than 4 days increased the risk of NEC [22–24]. Alexander et al. [24] reported that the incidence of NEC was 3 times higher in newborns who received prophylactic antibiotics for more than 10 days. A univariate analysis found that the failure of infants to achieve complete enteral feeding before the onset of NEC was associated with an increased probability of receiving surgical treatment. The reason may be that these infants were born at lower gestational ages, and immature intestinal development caused them to fail to achieve complete enteral feeding. In addition, compared with full-term infants, preterm infants have significantly delayed gastric emptying.

To date, clinical indicators are not reliable for predicting when NEC will occur or which children will develop NEC. Laboratory test results, such as increased or decreased white blood cell count, thrombocytopenia, metabolic acidosis, unstable glucose levels, and elevated C-reactive protein levels, can be observed in patients with NEC. However, these results still lack sensitivity and specificity [25, 26]. In our study, low platelet count, low serum HCO_3_^−^_,_ and increased standard base excess were associated with an increased probability of receiving surgical treatment in NEC patients. However, in the multivariate analysis, only low serum HCO_3_^−^ was identified as an independent predictor of an increased probability of undergoing surgery in NEC infants. Low serum bicarbonate can lead to metabolic acidosis, causing poor circulation or severe bicarbonate loss. Therefore, the abnormal serum HCO_3_^−^ and standard base excess in the sNEC group were not surprising because serum HCO_3_^−^ and standard base excess reflect the infants’ metabolic state.

Similar to other retrospective studies, this study has some inherent and unavoidable limitations. First, our study was based on a single center and might be affected by various biases. In general, multicenter and large-sample studies have less bias. Second, the data used in this study were collected from records in our electronic medical record system, and some important data might have not been recorded. Therefore, further prospective studies are needed to elucidate the reasons for the increased probability of surgical treatment in preterm infants with NEC.

## Conclusions

NEC is the most common and fatal gastrointestinal disease in preterm infants. There is an urgent need to make a timely and accurate diagnosis of NEC and to determine which children are at high risk of requiring surgery. The leading risk factors for an increased probability of receiving surgery in our study were lower gestational age, early occurrence of NEC after birth, hsPDA, and low serum bicarbonate. Our findings may support the clinician to identify infants with increased risk for sNEC, potentially leading to earlier surgical consultation, additional diagnostics, or even surgical intervention.

## Data Availability

The datasets used and/or analyzed during the current study are available from the corresponding author on reasonable request.

## Abbreviations

NEC: Necrotizing enterocolitis
mNEC: Medical necrotizing enterocolitis
sNEC: Surgical necrotizing enterocolitis
hsPDA: Hemodynamically significant patent ductus arteriosus
PDA: Patent ductus arteriosus
NSAIDs: Nonsteroidal anti-inflammatory drugs
